# Nano-mechanical characterization of the wood cell wall by AFM studies: comparison between AC- and QI™ mode

**DOI:** 10.1186/s13007-017-0211-5

**Published:** 2017-07-25

**Authors:** Kirstin Casdorff, Tobias Keplinger, Ingo Burgert

**Affiliations:** 1 Wood Materials Science, Institute for Building Materials, ETH Zürich, Stefano-Franscini-Platz 3, 8093 Zurich, Switzerland; 2 Applied Wood Materials, Empa-Swiss Federal Laboratories for Materials Science and Technology, Überlandstrasse 129, 8600 Dübendorf, Switzerland

**Keywords:** Atomic Force Microscopy, Wood, Spruce, Cell wall, Young’s Modulus

## Abstract

**Background:**

Understanding the arrangement and mechanical properties of wood polymers within the plant cell wall is the basis for unravelling its underlying structure–property relationships. As state of the art Atomic Force Microscopy (AFM) has been used to visualize cell wall layers in contact resonance- and amplitude controlled mode (AC) on embedded samples. Most of the studies have focused on the structural arrangement of the S_2_ layer and its lamellar structure.

**Results:**

In this work, a protocol for AFM is proposed to characterize the entire cell wall mechanically by quantitative imaging (QI™) at the nanometer level, without embedding the samples. It is shown that the applied protocol allows for distinguishing between the cell wall layers of the compound middle lamella, S_1_, and S_2_ of spruce wood based on their Young’s Moduli. In the transition zone, S_12_, a stiffness gradient is measured.

**Conclusions:**

The QI™ mode pushes the limit of resolution for mechanical characterization of the plant cell wall to the nanometer range. Comparing QI™- against AC images reveals that the mode of operation strongly influences the visualization of the cell wall.

## Background

Wood is a unique biological material with several levels of structural hierarchy from the nanometer level to the macroscale [1]. In particular the organization on the nanoscale, within the cell wall, is still under debate [2, 3]. The cell wall has been studied with numerous high resolution techniques like Transmission Electron Microscopy, TEM [4, 5], Scanning Near-field Optical Microscopy (SNOM) [6], or Atomic Force Microscopy (AFM) [3, 7–10]. A comparison of these studies shows that the observed nanostructural organization of the cell wall is strongly influenced by the used technique, and the sample preparation. Most of the previously cited studies have focused on the structural arrangement of the S_2_ layer and its lamellar organization, but also the other layers like the S_1_ and S_3_ are of importance for the mechanical behavior of the entire cell wall composite. TEM measurements on stained samples elucidated a transition zone between the S_1_ and S_2_ layers, called S_12_, where the lignin concentration decreases [11–14]. It is well known that in case of intra-wall failure, cell walls rupture predominately between the S_1_ and the S_2_-layer [14], however mechanical characterization of this mechanically highly relevant zone is missing.

State of the art imaging of secondary cell walls by AFM is in Resonant Contact mode [15, 16] and more common Amplitude Controlled mode (AC mode, an intermittent contact mode) of embedded cells [7, 17, 18]. Recent technical developments in the field of AFM allow to conduct a mechanical characterization in addition to topography studies, without sample embedding. Such multichannel AFM studies have first been applied for the characterization of primary walls [19, 20], and little has been done on secondary cell walls yet. Peakforce Quantitative Nanomechanics (PFQNM) has been used to image bamboo fiber cell walls [21] and to characterize the shape of cellulose microfibrils in primary plant cell walls in water using a very sharp probe with a curvature below 5 nm [19]. Adhesion force mapping was conducted to measure the inactivation of a freshly cut cell wall surface, and to study the influence of surface roughness and tip geometry [22, 23]. Recently, Arnould et al. [24] gave a proof of concept of high resolution force mapping on embedded flax fibers by studying the mechanical gradients using nanoindentation as a reference and additionally Muraille et al. [10] applied the protocol on poplar fiber cell walls, however without providing specific structural details.

The purpose of the present paper is to systematically explore the feasibility of using the Quantitative Imaging mode (QI™ mode), a force spectroscopy mode, which records a force–distance (FD) curve in every pixel, as a tool to characterize the wood cell wall with a specific focus on the transition from the middle lamella to the S_2_ layer on a cell wall cross-section of spruce. To be able to compare the structures imaged in QI™ mode to literature data, the cell wall was also scanned in AC mode. Thereby, structural information was revealed with high resolution, helping to better understand the underlying structure–property relationships of wood cell walls.

## Methods

### Spruce cube preparation

The cross-section of an air dried spruce cube (5 × 5 × 5 mm^3^) was prepared by means of a two-step polishing process. The plane of the sectioning was oriented perpendicular to the longitudinal fiber axis of the wood. A microtome with a steel knife (RM2255, Leica) was used to smoothen the surface under wet conditions. Following the protocol of Keplinger et al. [6], an ultramicrotome (Ultracut, Reichert-Jung) equipped with a Diatome Histo diamond knife was used to polish. The typical root-mean-square roughness of a 1 × 1 µm^2^ cross-section was less than 1.4 nm. The microfibril angle (MFA) was measured on a spruce sample, cut out of the same strip of wood to gain samples from longitudinally matched positions, by wide angle X-ray scattering on the longitudinal-transverse plane. A MFA of 6° was recorded, which points to a mature wood probe.

### Quantitative imaging AFM

AFM imaging was performed using a NanoWizard 4 (JPK Instruments AG) in QI™ mode under controlled climatic conditions (temperature 20 °C, humidity 65%). As a cantilever, a non-contact cantilever (NCHR, Nano World, resonance frequency 320 kHz) with a silicon probe was used. The cantilever was calibrated with the contact-free method for a beam shaped cantilever by giving the environmental conditions and cantilever dimensions [25]. The force constant was calibrated to be 30 N m^−1^ (±1 N m^−1^, n = 5) and the deflection sensitivity was determined to be 24 nm V^−1^ (±1 nm V^−1^, n = 5). The setpoint of the measurement was defined according to the cantilever stiffness (60 nN) and the z-length (50 nm) and the pixel time (12 ms) were set fixed, to ensure a similar velocity for each measurement. This resulted in an extend rate of 62.5 kHz (the extend rate controls the speed of the movement on the extend part of the FD curve), and an extend speed of 10.42 µm s^−1^.

The software extension Advanced QI™ mode was used to have full access to all FD curves. The mapping resolution was chosen to be 256 × 256 pixels and the scan size was set between 10 × 10 µm^2^ and 1 × 1 µm^2^ (theoretical resolution limit 3.9 nm). The AFM was operated in z-closed loop, therefore the nonlinearity and hysteresis of the piezo were corrected during the movement (channel: height measured) and FD curves had a constant speed. The baseline was adjusted to correct for any changes of the setpoint.

**Fig. 1:**
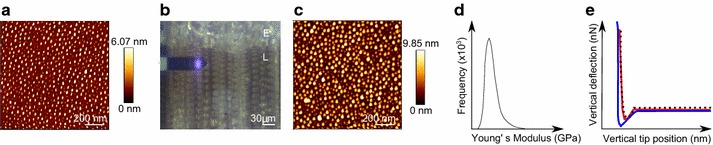
Basic flow chart of the measurement routine. **a** A tip and resolution test specimen is scanned. **b** The wood sample is scanned, *E* earlywood, *L* latewood. **c** The tip and resolution test specimen is scanned again. **d** A stiffness test specimen is scanned and the tip radius is fitted to the stiffness of the polystyrene. **e** The Young’s Modulus can be calculated for the wood sample by fitting (*dotted black line*) the *trace curve* (*red*) with the Derjaguin–Müller–Toporov model (*blue*, *retrace curve*)

Prior and after a cell scan, the tip resolution at 0° and 90° scan direction was checked using a test specimen (Product No. 628-AFM tip and resolution test specimen, Pelcro, TED PELLA INC.). The tip radius was fitted according to the stiffness of polystyrene (2 GPa) after scanning a polymer test specimen (PS-LDPE-GS, Veeco Metrology Group) (Fig. 1).

The data was analyzed in the JPK image processing software (JPK Instruments AG). A line fit was applied to correct the height measure image. To avoid characteristic shadows around high objects, the areas with elevated features were excluded from the line fit by a region of interest. As a second step possible default lines were replaced with the average between the adjacent lines. Force curve batch processing was performed in the following order on the extend curve: (1) Calibration of V-deflection (sensitivity and spring constant). (2) Smoothing of force data. (3) Baseline subtraction for offset and tilt by defining the fit range from 100% to the snap in, normally around 10%. (4) Contact point determination: Calculates vertical tip position, corrects the height signal for the cantilever deflection. (5) The Young’s Modulus can be calculated from the slope of the force curve by applying a contact mechanics model. As suggested by the suppliers, in PFQNM (Bruker) the Young’s Modulus is determined with the Derjaguin–Müller–Toporov (DMT) model of elastic contact on the retract curve [26], whereas in QI™ the extend curve is fitted to avoid any influence from plastic deformation. The Young’s Modulus was calculated with the DMT model, assuming a Poisson’s ratio of 0.4 (adapted from Gibson and Ashby [27]), and the respective radius of the cantilever. The quality of the fit was inspected visually for a typical FD curve. Due to the high amount of curves generated within one image, the fit cannot be optimized for each curve, but the weight of a false processed curve is therefore limited. The results were plotted in a histogram revealing the distribution of the data.

To remove cutting artefacts, the image data can be corrected with a two-dimensional fast Fourier transform (FFT) using Gwyddion 2.47.

### AC mode AFM

In AC mode the mapping resolution was increased to 512 × 512 pixels, taking the equivalent time as the QI™ image. The full z-range of 15 µm was used. For the calibration of the cantilever the setpoint amplitude was selected to be around 70%. The approach was performed with constant velocity and baseline update at the starting point. The gain parameters were optimized in respect to the offset of the trace and retrace line in the oscilloscope. The channels were post-processed according to the height measured image described for the QI™ mode.

## Results and discussion

### Imaging cell walls in QI™ mode

**Fig. 2:**
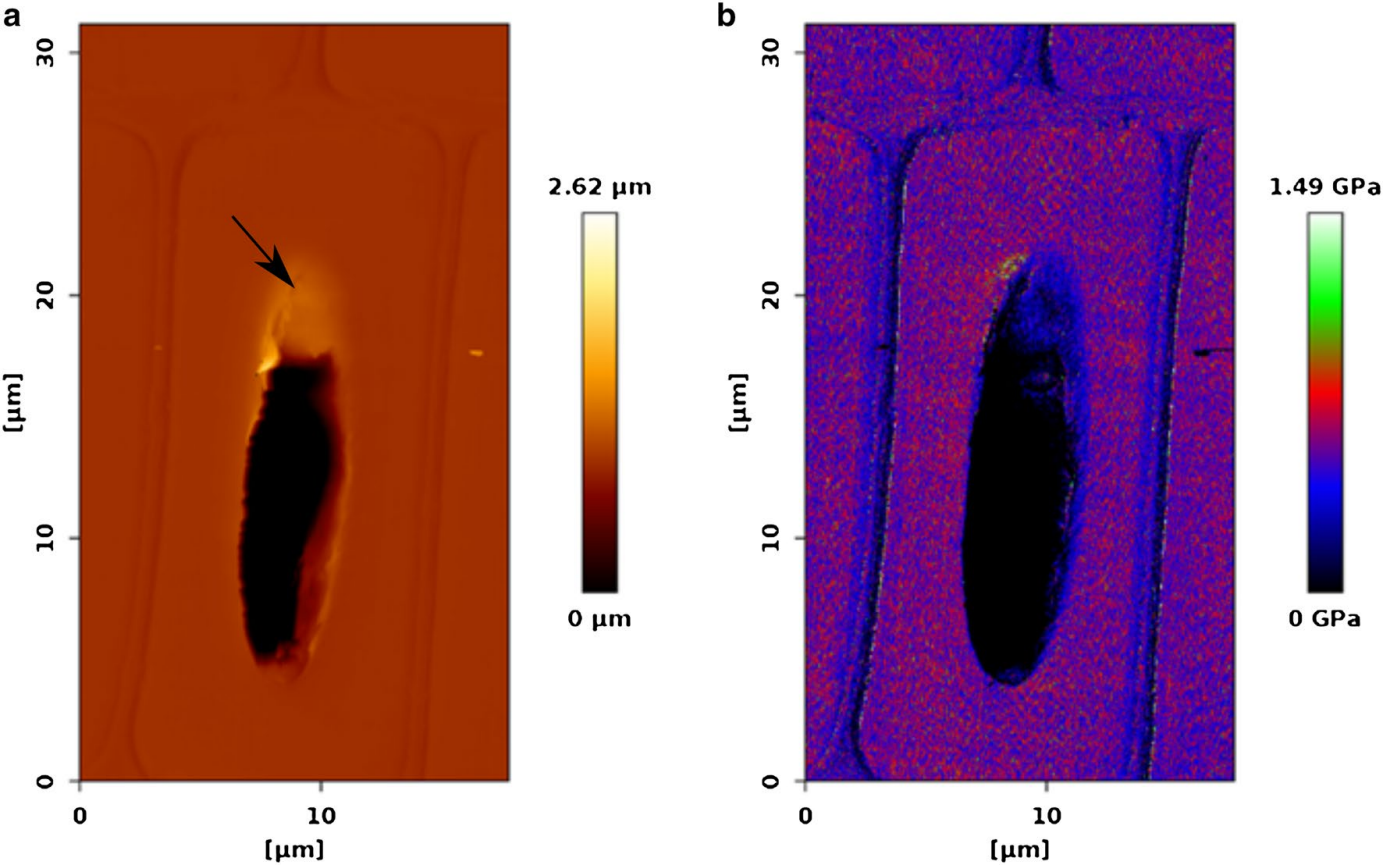
**a** Height image and corresponding, **b** Young’s Modulus image of a whole latewood cell (512 × 512 pixels). The radial direction runs from *left* to *right*. The *black arrow* point to an inclination at the lumen/cell wall interface

By using the QI™ mode it is possible to scan over a whole unembedded cell through the lumen, because for FD curves the cantilever only moves the z-length that was set, in this case 200 nm (Fig. 2). Although the resolution of such a large scan (18 µm × 31 µm) is reduced in x-direction to 35 nm and in y-direction to 60 nm, stiffness changes between the stiff S_2_ and the softer middle lamella and S_1_ layer regions can clearly be seen in the Young’s Modulus image (Fig. 2b). Furthermore, the quality of the cut could be judged based on the visible inclination at the lumen/cell wall interface in the height image (Fig. 2a, black arrow).

**Fig. 3:**
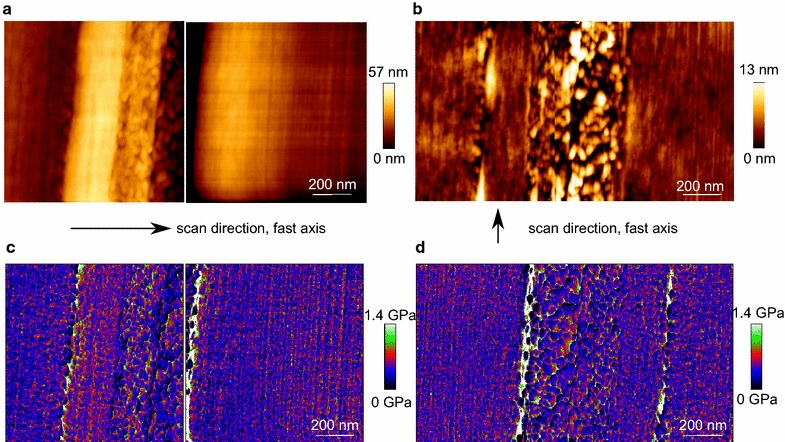
Height- and Young’s Modulus image of a compound middle lamella scanned at two perpendicular directions. **a**, **c** 0° scan direction perpendicular to the structures and **b**, **d** 90° scan direction in line with the structures. The cutting direction is perpendicular to the compound middle lamella

The middle lamella reveals no clear transition to the adjacent primary wall. Therefore, the middle lamella and both adjacent primary walls are termed compound middle lamella, CML. The height images of the CML appear different depending on the scanning direction, which can be perpendicular, or in line to the cell wall layers (Fig. 3). This is a typical artefact in AFM imaging when scanning oriented structures [28], and needs to be considered when comparing different positions in the cell wall. Interestingly, the corresponding Young’s Modulus images are not affected by the scanning direction.

### Comparison of QI™- and AC mode

In QI™ mode the algorithm of the tip motion measures a FD curve in every pixel, with a defined setpoint [29]. Thereby, besides topological information the sample can be characterized mechanically with a high spatial resolution at high speed. There is no xy movement during the FD curve recording, which ensures a measurement under constant velocity. In AC mode the scanning cantilever is oscillating at a kilohertz range frequency, and as an additional channel the phase image can be displayed. The lock-in-amplifier measures a phase shift between the drive signal and the cantilever movement in dependency of the tip-sample interaction, including mechanical information, adhesion, and dissipation of cantilever energy [30]. In order to compare the imaging mode QI™ to the state of the art applied AC mode, we choose to scan with both modes the area of transition from the compound middle lamella to the S_2_.

### QI™ mode

**Fig. 4:**
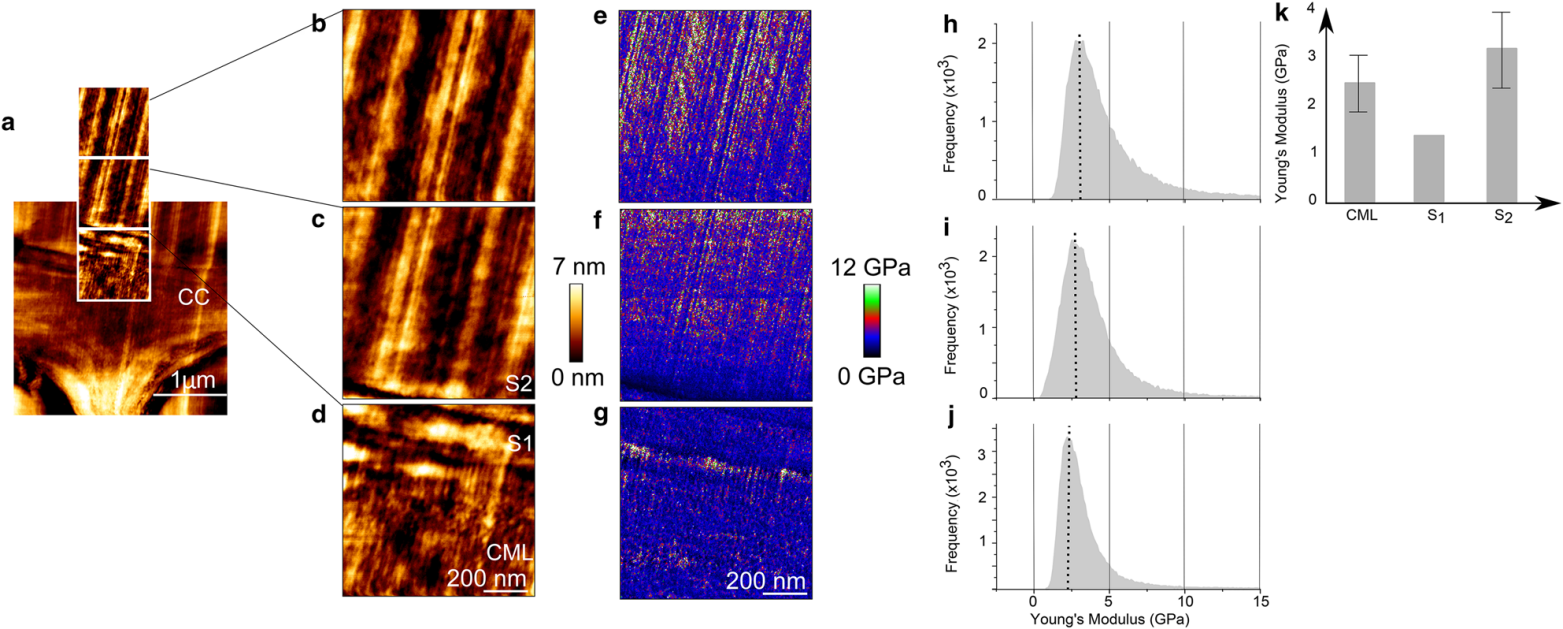
**a** Overview of the cell corner (3 × 3 µm^2^) imaged in QI™ mode. **b**–**d** 1 × 1 µm^2^ height-, corresponding, **e**–**g** Young’s Modulus images and **h**–**j** histograms. **k** Bar chart summarizing the Young’s Modulus values of the distinct cell wall layers. The transition was scanned from **b** the S_2_ to **d** the compound middle lamella. All images are set to the same scale, the radial direction points from *right* to *left*. The histograms show the distribution of Young’s Modulus over the 1 × 1 µm^2^ image. *CC* cell corner, *CML* compound middle lamella, *S*_*1*_, *S*_*2*_ different layers of the secondary cell wall

Figure 4a shows an overview image of a cell corner and Fig. 4b–d display a zoom into the transition zone from the middle lamella to the S_2_-layer. The vertical straight lines in the images of the S_2_ are cutting artefacts that result from imperfections of the diamond knife. The CML is buildup of an isotropic structure consisting mainly of lignin, that is assumed to organize in a self-assembly process [31, 32]. After the CML follows a narrow zone of approximately 100 nm, most presumably the S_1_. While the S_1_ has a comparable denser structure, the S_2_ appears as a woven network. The typical lamella structure of the S_2_ cannot be clearly visualized because, (1) cutting artefacts may overly the lamella, (2) the scanned area might be too close to the CML, and/or (3) microfibrils are strictly parallel aligned [2].

The Young’s Modulus of the S_1_ was determined by selecting a rectangle of maximum size inside the cell wall layer and calculating the root mean square value (Fig. 4g). Due to its large MFA, typically 70–90°, the S_1_ possesses a lower stiffness than the S_2_ (Fig. 4e) [12]. Surprisingly, the S_1_ stiffness is with 1.3 GPa lower than the CML (data obtained from a region of interest comprising approximately 5625 FD curves). This might be explained by the different textures of the loose CML and the denser S_1_ layer, leading to artificially higher peak values in the CML. This indicates that due to the specific surface- cantilever tip interactions the structural patterns of the cell wall layers affect the stiffness values and that the stiffness ratios between the cell wall layers can be altered.

In the corresponding stiffness images and histograms it can be seen, how the Young’s Modulus changes from a narrow Gaussian distribution mean value of around 2.5 ± 0.6 GPa in the CML (Fig. 4j) to a broader distribution around 3.2 ± 0.8 GPa in the S_2_ layer (Fig. 4h, for a comparison the values are summarized in Fig. 4k, data obtained from one image comprising approximately 65536 FD curves). The histogram distribution of the S_2_ might be larger, because of being more heterogeneous in terms of biopolymer composition compared to the middle lamella, which is mainly composed of lignin [33]. The above-mentioned transition zone between the S_1_ and S_2_, called S_12_, where the lignin concentration decreases [11, 12], can be visualized in the QI™ mode over a length of 2 µm by an increase in Young’s Modulus from the S_1_ to the S_2_ (Fig. 4f,i). Depending on the depth information measured by AFM, the increase in Young’s Modulus can arise from (1) the stiff cellulose microfibrils oriented with a small MFA, (2) the change in the surface structure due to a change in MFA, and/or (3) the change in composition of the different cell wall layers. Further experiments on composite model systems, with defined MFA on a similar length scale than wood, are required to make a certain statement about the parameters influencing the measured Young’s Modulus. Certainly, very low stiffness values for the wood cell wall, respectively its individual layers were measured in comparison to mechanical data obtained by tensile tests [34–36] and nanoindentation [37, 38]. The entirely different loading conditions and test geometries in micro- and macroscopic tests do not allow for a direct comparison with the AFM data. Since the cell wall stiffness values obtained by nanoindentation and those derived from micro- and macroscopic tests are in the same range, a focus is laid on a comparison with values measured by nanoindentation, which was recently discussed by Arnould and Arinero [39].

The contact mechanism of the probe of a nanoindenter considerably differs from an AFM tip in terms of geometry and indentation depth, which results in different interactions with the wood surface, because of the anisotropy of wood [39, 40]. Comparing stiffness values obtained by different AFM modes, Arnould et al. [24] applied PFQNM on a flax fiber with a setpoint of 200 nN on the retrace curve. For the S_1_ and CML they calculated a Young’s Modulus of 7 GPa. For the S_2_ they obtained values from 13 to 18 GPa, which is comparable to the values they measured with a nanoindenter. Muraille et al. [10] applied PFQNM on a transverse section of poplar plant fiber cell wall with a setpoint of 600 nN. The indentation moduli of the CML, S_1_ and S_2_ were averaged 17, 21, and 26 GPa. In our measurement the penetration depth is very small as at a setpoint of 60 nN, the penetration is around 5 nm. Thereby, only surface properties are determined and no plastic deformation takes place. The setpoints mentioned in the two studies before are three to ten times higher than in our measurement, therefore it is assumed that also the penetration depth was larger. As wood is a viscoelastic material, the scan speed of the measurements also needs to be taken into account, when comparing the obtained values. In QI™ mode the supplier suggests to use the trace curve, whereas in PFQNM it is suggested to use the retrace curve, for the fitting routine of the contact model. In our measurements, the slope of the retrace curve was too steep to be fitted. The trace curves could be fitted with the Hertz- [41] and the DMT model [26], with the DMT model giving higher stiffness values. The roughness of the microtome polished surface was very small. For the CR-AFM and the PFQNM measurement of Arnould et al. [24] the Young’s Modulus increases from the CML to the S_2_ by a factor of two. Although the values in our measurements were smaller, the same tendency was found.

### AC mode

**Fig. 5:**
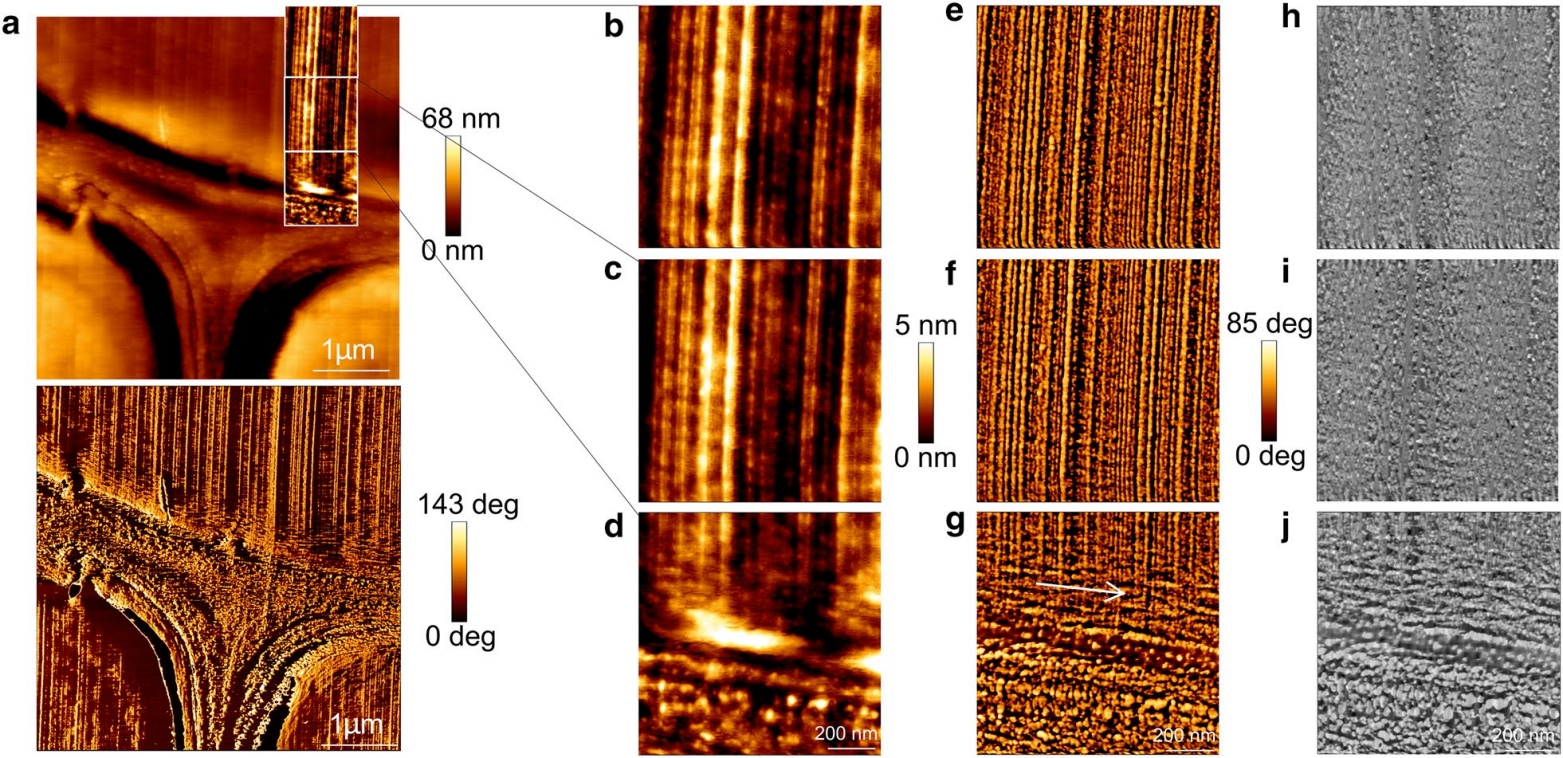
**a** Overview of the cell corner (4.5 × 4.5 µm^2^) imaged in AC mode. **b**–**d** 1 × 1 µm^2^ height-, corresponding, **e–g** phase- and **h**–**j** fast Fourier transformed images. The transition was scanned from **b** the S_2_ to **d** the compound middle lamella. All corresponding images are set to the same scale, the radial direction points from *right* to *left*. *No color scale* is shown for the FFT phase image as the image information could not be transferred during the FFT. The *white arrow* points to the directionality of the S_12_ layer

To compare the QI™ mode with the AC mode, Fig. 5 displays the transition from the CML to the S_2_ imaged in AC mode. The height images in the two modes are comparable (Fig. 5b–d). The phase image shows lignin in the CML as ellipsoid structures and the adjacent S_1_ layer possess a low phase signal (Fig. 5e–g). In phase contrast images, typically darker areas can be correlated with regions of lower stiffness [30]. By applying a FFT the directed cutting artefacts can be selectively removed from the phase image, thereby the structural change from the S_1_ layer to the S_2_ layer becomes more obvious (Fig. 5h–j). The transition zone S_12_ shows a parallel orientation to the CML (white arrow), whereas the S_2_ has a granular structure. The transition zone visualized in the phase image is smaller than the one from the Young’s Modulus image, as the Modulus image is more sensitive for detecting changes in mechanical properties.

Fahlén and Salmén [17] visualized individual cellulose aggregates ranging from 10 to 30 nm in the S_2_ layer by phase imaging oriented with a regularity that was interpreted as an additional lignin pattern. Here, we fitted the structures in the FFT of the S_2_ and they lay in the same size range compared to the one observed by Fahlén and Salmén [17]. The lignin structures of the CML had an ellipsoid shape ranging from 30 to 60 nm diameter. Due to the influence of the cutting artefacts a clear lamellar structure in the S_2_ could not be detected.

### Comparison fast Fourier transform

**Fig. 6:**
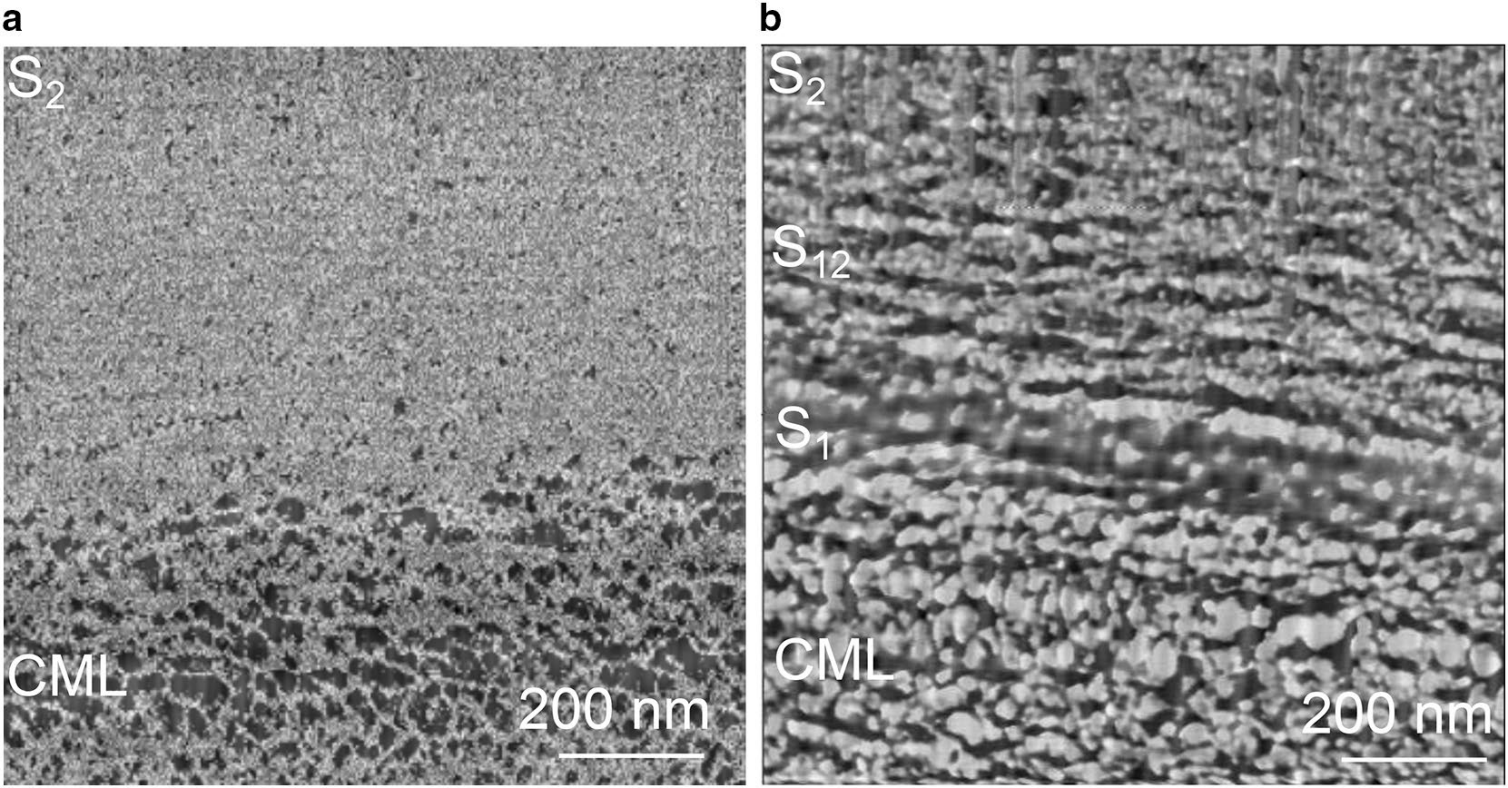
Comparison of the fast Fourier transform (FFT) image scanned by **a** QI™ mode and **b** AC mode (enlargement of Fig. 5j). *No color scale* is shown as the image information could not be transferred during the FFT. *CML* compound middle lamella, *S*_*1*_, *S*_*12*_, *S*_*2*_ different layers of the secondary cell wall layer

In Fig. 6 two enlarged FFT corrected images of the transition between the CML and S_2_ scanned in QI™- (Fig. 6a) and AC mode (Fig. 6b) can be seen. After FFT processing the variability of the Young’s Modulus values could be reduced to two colors (the lighter, the stiffer), thereby the gradient information is lost. One can only distinguish the CML from the S_2_. The CML appears as a porous network, whereas the S_2_ seems to be dense. The phase image has a twice as high resolution, therefore it appears sharper. The CML gives the impression of a negative of the Young’s Modulus image and the S_2_ has a granular appearance. The phase shift is proportional to the stiffness, but phase contrast interpretation is not as straight forward like the analysis of FD curves, due to the contributions from contact area, viscoelastic properties, and capillary forces [30].

## Conclusions

In conclusion, QI™ mode gives the opportunity to mechanically characterize the different secondary cell wall layers on the nanometer level by obtaining FD curves that can be analyzed with a mechanical model. Although too low stiffness values are measured, it outperforms mechanical characterization by nanoindentation in terms of resolution, and provides a more distinct image of stiffness distribution than what can be obtained by phase contrast imaging in AC mode.

 The imaging mode QI™ is on the one hand very robust, as it can scan over whole unembedded cells, and on the other hand it is sensitive enough to detect small changes, as can be seen from the visualization of the transition zone S_12_. The nanostructure of wooden cell walls is just at the beginning to be characterized mechanically. Comparative studies are needed to unravel the influence of never dried wood, different cell types, or different species on the mechanics. Further insights into the cell wall assembly can be used to understand the localization of chemical modifications within wood beyond the resolution limit of commonly used techniques [42], or to set up reliable computational models on the microscale [43–45].

## Abbreviations

AFM: Atomic Force Microscopy
CR-AFM: contact resonance AFM
DMT: Derjaguin–Müller–Toporov
FD curve: force–distance curve
FFT: fast Fourier transform
JKR: Johnson–Kendall–Roberts
MFA: microfibril angle
PFQNM: Peakforce Quantitative Nanomechanics
QI™: Quantitative Imaging
